# Combining participatory and socioeconomic approaches to map fishing effort in small-scale fisheries

**DOI:** 10.1371/journal.pone.0176862

**Published:** 2017-05-09

**Authors:** Lauric Thiault, Antoine Collin, Frédérique Chlous, Stefan Gelcich, Joachim Claudet

**Affiliations:** 1 National Center for Scientific Research, PSL Research University, CRIOBE, USR 3278 CNRS-EPHE-UPVD, PSL Research University, Perpignan, France; 2 Laboratoire d'Excellence CORAIL, France; 3 Museum National d’Histoire Naturelle, PALOC, UMR 208 MNHN-IRD, Paris, France; 4 Center of Applied Ecology and Sustainability, Departamento de Ecología, Facultad de Ciencias Biológicas, Pontificia Universidad Católica de Chile, Santiago, Chile; 5 PSL University—EPHE, CNRS Prodig, Dinard, France; Leibniz Center for Tropical Marine Ecology, GERMANY

## Abstract

Mapping the spatial allocation of fishing effort while including key stakeholders in the decision making process is essential for effective fisheries management but is difficult to implement in complex small-scale fisheries that are diffuse, informal and multifaceted. Here we present a standardized but flexible approach that combines participatory mapping approaches (fishers’ spatial preference for fishing grounds, or fishing suitability) with socioeconomic approaches (spatial extrapolation of social surrogates, or fishing capacity) to generate a comprehensive map of predicted fishing effort. Using a real world case study, in Moorea, French Polynesia, we showed that high predicted fishing effort is not simply located in front of, or close to, main fishing villages with high dependence on marine resources; it also occurs where resource dependency is moderate and generally in near-shore areas and reef passages. The integrated approach we developed can contribute to addressing the recurrent lack of fishing effort spatial data through key stakeholders' (i.e., resource users) participation. It can be tailored to a wide range of social, ecological and data availability contexts, and should help improve place-based management of natural resources.

## Introduction

Small-scale fisheries, which have been defined at those “*traditional fisheries involving fishing households (as opposed to commercial companies)*, *using relatively small amounts of capital and energy*, *relatively small fishing vessels (if any)*, *making short fishing trips*, *close to shore*, *mainly for local consumption”* [1], provide an iconic example of the intricate links between people and nature. Food and capital accumulation through fishing and selling of marine products are important for food security and poverty alleviation, especially in developing countries [2–4]. Less tangible benefits such as well-being, and individual and collective cultural identity also make small-scale fishing strongly embedded in the lifestyle of many fishing communities [5–8]. Their characteristics, compared to large-scale fisheries, have often been advanced by academics as key aspects of their sustainability [9,10]. However, in most countries, issues including conflicts with industrial fisheries [11,12], open-access to fisheries [13] or use of destructive fishing methods and poverty traps [14–16], have led many small-scale fisheries to be exploited beyond sustainable levels [17]. Securing fisheries’ and livelihoods’ sustainability, and preventing or escaping from social-ecological traps can only be achieved with sound fisheries management [18,19].

Management of small-scale fisheries requires knowledge to make decisions about where, when, to whom and to which extent resources should or should not be allocated [20–22]. One of the overarching challenges of current fisheries science is that data related to the human-nature interactions are difficult to integrate into tools that can effectively guide decision-making [23,24]. Albeit advocated as a critical input for policymakers and managers [25], the spatial distribution of resource use (hereafter referred to as fishing effort) is no exception due to the often diffuse and informal nature of the fisheries, the variety of motivations to fish among individuals (e.g., to eat, to sell and/or for pleasure), the diversity of strategies regarding gear, habitats and species caught, and a common lack of human, technical and financial resources for data collection and processing [26]. Such complexity in assessing spatial distribution have made conventional quantitative assessments of fishing effort such as fleet registers, catch declarations, sales notes and individual tracking from vessel monitoring systems relatively uncommon in small-scale fisheries (but see [27] for counterexample).

Going beyond conventional fisheries assessment methods requires alternative approaches that better incorporate the human dimension while coping with the inherent complexity of small-scale fisheries [28,29]. At the local scale, academics and practitioners have already begun to integrate social components into spatial assessments of fishing effort [30,31]. Such methods include interview data and quantitative participatory processes to better understand fishing intensity at particular sites [32,33], individual or collective description of the value of fishing areas [34,35], focal follows [36] and self-reporting diaries [37]. These approaches have the advantage of generating a great amount of spatial information about linked provisioning and cultural services [38], can yield information about fishing practices at high temporal and spatial resolution, and facilitate gathering of additional data such as local ecological knowledge. However, for this information to scale in coverage and produce reliable fishing effort estimates at the fishery level, these approaches require large sample sizes and appropriate sampling designs, which are rarely achieved due to logistical constraints (but see [37,39]). Therefore, obtaining reliable information on fishing effort through active participation of fishers (from now on referred to as direct participatory approaches) may be only applicable if some particular conditions are met, or if significant financial, human and time investments are committed, which unfortunately is generally not the case in small-scale fisheries.

When participatory methods are difficult to implement or when the outputs are uncertain, national socioeconomic statistics (hereafter referred to as population censuses) and other sources of large-scale, non-fishery-related, information, may represent a key contribution to fisheries spatial pattern assessments. In the same way that taxonomic or environmental surrogates are used to depict the spatial patterns of other–unknown–aspects of biodiversity [40], socioeconomic approaches based on social surrogates can help to fill the lack of fisheries data in small-scale fisheries. Previous studies have used proxies based on distance to fishing ports or accessibility points [41], population density [42] or number of boats [43,44] to predict the spatial allocation of the fishing effort. However, relying only on such fairly coarse proxies for place-based management purposes can be misleading as it assumes that fishers’ spatial behavior is random and only driven by the distance to their place of departure (e.g., port, settlement, accessibility point), which is unlikely to be the case in most contexts [33,45].

Here, we propose a standardized but flexible approach that addresses the difficulties of obtaining accurate spatial fishing effort allocation data in small-scale fisheries, by linking participatory and socioeconomic approaches to model fine-scale fishing effort distribution. The approach requires the combination of fine-scale representation of fishers’ spatial preference for fishing grounds (estimated through a participatory approach) with fishing capacity (estimated through a socioeconomic approach). We tested our integrated approach in the context of the small-scale coral reef fishery of Moorea, French Polynesia, which shares a number of important features with an array of other coral reef small-scale fisheries [46].

## Methods

### Theoretical approach for mapping relative fishing effort

**Fig 1:**
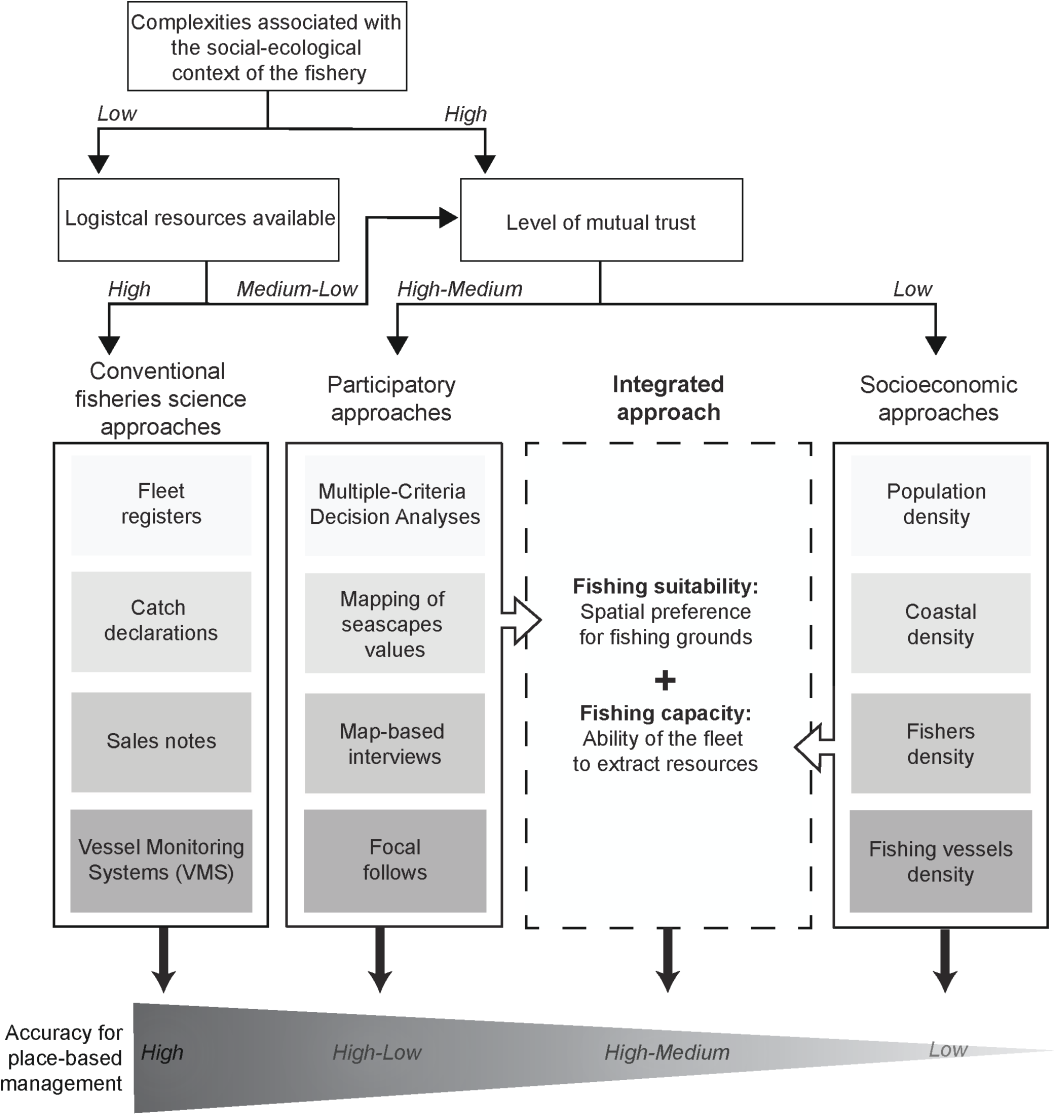
Conceptual flowchart for selecting the best approach to map fishing effort according to availability of three critical factors to be considered by practitioners, namely the complexity of the social-ecological context, the availability of human and financial resources and the degree of cooperation possible with local fishers (i.e., mutual trust level). Techniques commonly used in each type of approach are indicated in boxes. The accuracy of each approach for place-based management (i.e., reliability of the gathered information, level of accuracy/resolution achieved and add-on information gathered during data collection) is provided. Although providing the most accurate estimates of fishing effort, fisheries approaches are unlikely to work in most small-scale fisheries due to the inherent complexity of the social-ecological context and the recurrent lack of logistical resources. Depending on the degree of participants’ engagement in the participatory process, information gathered through participatory approaches can be either highly (e.g., using self-reporting diaries and map-based interviews) or moderately (e.g., collective mapping of seascapes values and weightings of spatially-explicit criteria through Multiple-Criteria Decision Analysis) relevant for place-based management. Socioeconomic approaches rely on the extrapolation of social surrogates such as total or coastal population density, fisher or vessel density and may therefore fail to represent fine-scale patterns of the fishing effort (i.e., low accuracy for place-based management). The approach we present here proposes to combine the ability of participatory approaches to map fishers’ spatial preference (i.e., fishing suitability) with the power of socioeconomic approaches to estimate the fishery’s ability to extract resources (i.e., fishing capacity) and create fine-scale information on the spatial distribution of the fishing effort.

Spatial fishing effort allocation is considered here at the fishery level in terms of overall patterns of distribution. It is analyzed considering two components, namely (i) the fishing suitability and (ii) the fishing capacity (Fig 1). Here, fishing suitability refers to the suitability of fishing grounds. It can be represented spatially using quantitative participatory approaches involving direct or indirect representation of fishers’ spatial preference, depending on the ability of practitioners to engage fishers in the participatory process. Fishing capacity designates the overall ability of the fishery to extract resources in a given area. Socioeconomic approaches can provide large-scale and continuous estimations of this aspect of fishing effort, but remain too coarse to be used at local scales. The rationale of this approach is therefore to combine the in-depth knowledge from the fishing suitability analysis and the broader scale fishing capacity analysis as a way to scale the coverage of participatory methods to determines where the fishing effort concentrates.

### Mapping fishing suitability

A wide array of participatory approaches have successfully described fishers’ spatial preferences at high resolution through a variety of direct quantitative mapping techniques [47,48]. However, such direct mapping approaches require access to and a high degree of cooperation with local fishing communities and hence rely on deeply rooted, and notoriously hard to control, factors such as historical (dis)trust between scientists and fishers, organizational capacity of the fishers and accuracy of fishers’ answers. They are also difficult (and expensive) to scale in coverage and enable statistical generalization to the overall fishery.

In contexts where direct participatory mapping methods are difficult to conduct, indirect approaches that quantify the relative importance (weight) of criteria involved in fishing ground selection (e.g., habitat, depth or marine traffic activity) can facilitate the mapping of fishing suitability. Mapping spatially-explicit criteria can be achieved in many ways, depending on the criteria considered, the logistical resources and biophysical context. For instance, acoustic systems can yield high-resolution images of the seabed but are costly and not suitable for large areas [49,50]. Remote sensing has proven accurate and cost-effective for mapping habitat-related criteria (e.g., geomorphologic zones and substrate types) [51]. Finally, other methods requiring less technologies are suitable to spatially represent cost-related criteria (e.g., distance to nearest port) [52]. It also requires appropriate methods to consider uncertainties and multiple value judgments at stake in the decision-making process. A variety of Multiple-Criteria Decision Analysis (MCDA) methods has been developed for solving multiple-criteria decision-making problems and computing criteria weights. Although criteria weights can be directly assigned by the decision-maker (e.g., weighted ranking method), it is acknowledged that using ranks to elicit scores through mathematical formulas is more reliable because decision-makers are more confident about the ranks of some criteria than their weights [53]. Well-accepted weighting methods based on rankings include the ratio method [54], the Analytic Hierarchy Process (AHP; [55])and the Measuring Attractiveness by a Categorical Based Evaluation Technique (MACBETH; [56]) (for comparison of these methods see [57,58]).

### Mapping fishing capacity

The literature on systematic marine conservation planning (which is closely linked to fisheries management and marine spatial planning literatures [59]) provides a classification through which various levels of fishing capacity resolution can be structured hierarchically by progressively adding more detailed information [42,60]. In its simplest form, fishing capacity can be estimated and mapped as the population density extrapolated onto the water to a given distance of influence using density decay. This straightforward approach relies on the implicit assumption that the proportion of fishers is evenly distributed within the study area, which is not the case in most contexts [61]. One way of gaining accuracy is to restrict the extrapolation process to the coastal population when fishers live close to the shore. Nevertheless, the latter approach still assumes that the fisher population is proportionately spread within the overall population. A step forward is hence to consider the number of fishers based on socioeconomic characteristics (i.e., using population censuses, which are collected from the entire population). In the case where such information is available, fishers may be identified based on their principal and secondary declared livelihood activities. Once fishers are identified and located, fishing capacity can be estimated by (linear or other) distance function of the estimated number of fishers to home ports, accessibility points or markets rather that the entire coastline. An even finer resolution can be added by also integrating boat ownership. Fishing capacity can thus be approximated as a function of fishing vessels density within a radius that depends on the type of fishing vessel.

### Mapping relative fishing effort

Fishing suitability determines where the fishing capacity is distributed. Hence, fishing suitability can be used as a weighting factor of the fishing capacity (or its transformation) to create a map of relative fishing effort.

### Application to a case study

#### Moorea’s coral reef fishery

**Fig 2:**
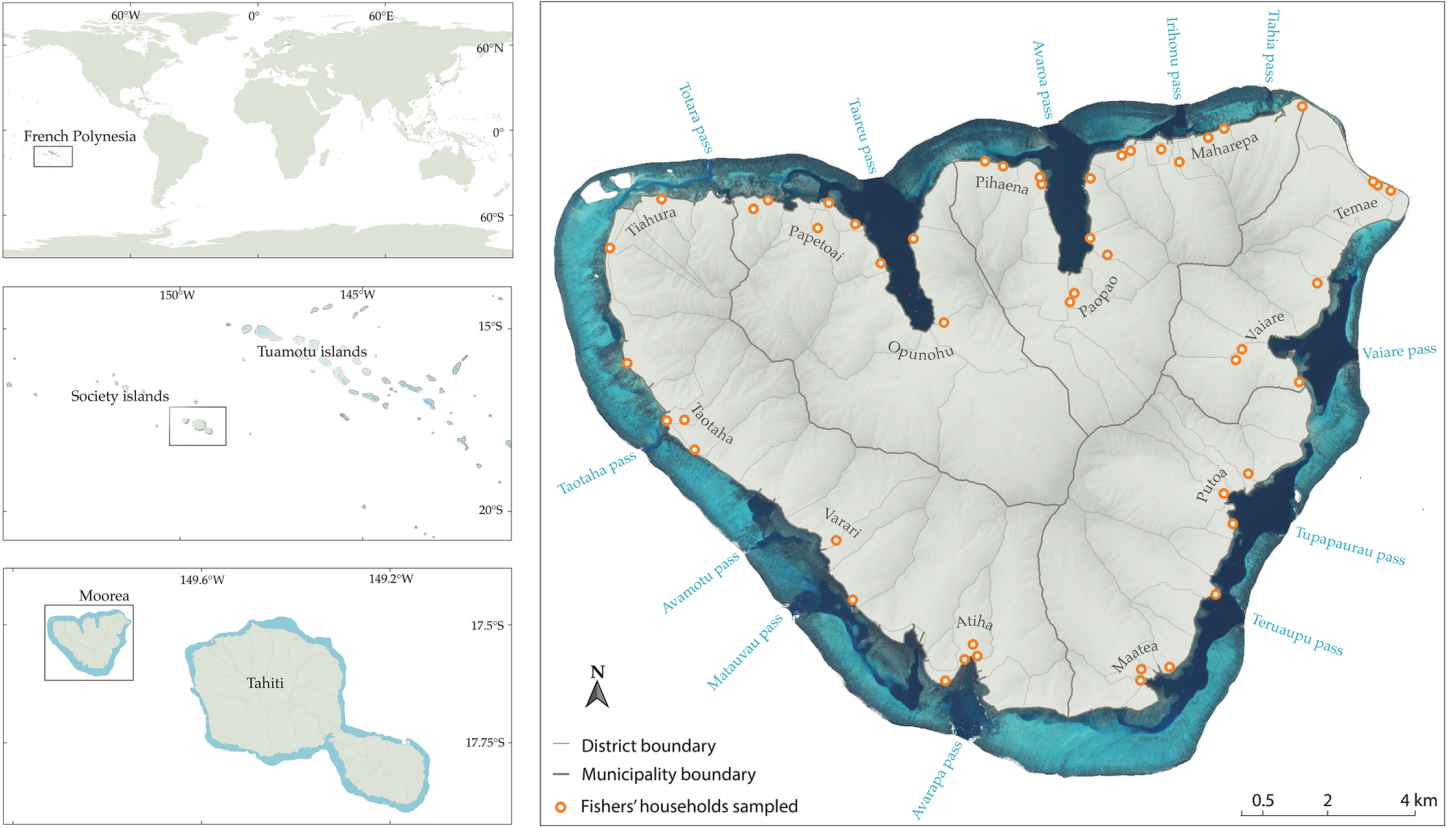
Map of Moorea Island, French Polynesia. Orange circles represent the location of households surveyed to quantify criteria and sub-criteria weights. Thick lines denote municipality boundaries and thin lines district boundaries. Key place names are indicated either in blue (reef passages) or in black (villages).

We applied this approach to assess the small-scale fin-fish fishery of Moorea island, French Polynesia (Fig 2), which is acknowledged as very challenging to assess [46]. More than 3/4 of Moorea's land area consists of uninhabitable volcanic peaks. As a consequence, the 17,000 inhabitants are mainly concentrated along a coastline of just over 60 km long and ancient villages occupying small valleys [62]. This particular arrangement is probably an important factor explaining why the marine environment and its use, mostly fishing, are still strongly embedded in the livelihood and lifestyle of the local population despite a recent switch from a rural to an urban economy (due to the proximity to the main island Tahiti and establishment of several hotels; [63]). 23% of the adult population still derives some or all of its subsistence and/or income from marine resources, with 35% of households engaged in a fishery-related activity [62]. Consumption surveys conducted in 136 households have highlighted the critical importance of unreported catches due to self-consumption and shares among family or other village members into the total catches [64]. Catches that still go through conventional sales channels remain hard to assess due to the absence of a market on the island. Instead, fishes are sold by the roadside, generally close to fishers’ houses, making the entire coastline a potential landing/selling area. Such ubiquitous, diffuse, and atypical features make landing surveys and direct observation unsuitable to provide reliable information regarding spatial patterns of the fishery [46].

A spatially-explicit management plan (*Plan de Gestion de l’Espace Maritime*, *PGEM*), including a network of eight permanent Marine Protected Areas (MPAs), was officially established in 2004, although its actual implementation was only achieved in 2007. During the ten year long planning process prior to the implementation of the management plan, fishing grounds and fishing effort were not properly considered due to the absence of adequate data during this period [65]. As a consequence, a significant number of fishers still question the legitimacy of this management plan today, which may–at least partly–explain the weak compliance of users with fishing regulations [66] and the unclear effect of MPAs on marine resources [67]. In addition, previous planning processes have created distrust among stakeholders and widespread participatory approaches, such as direct mapping of fishing grounds or self-reporting diaries, are unlikely to succeed (but see [68] for application of a direct participatory approach to map general fishing areas at three locations around Moorea).

#### Mapping fishing suitability

In order to map fishing suitability of the overall fishery, we applied an indirect mapping method following five main steps. First, we first identified biophysical and cost-related criteria (and sub-criteria) considered by local fishers when making decisions about where to go fishing in the long run based on a literature search, six key informant interviews and five pilot interviews conducted with fishers. They included Distance to the shore (0-400m, 400–1,000m and >1,000m), Depth (0-3m, 3-8m and >8m), Distance to the closest pass (0-250m, 250–1,000m and >1,000), Slope of seafloor (flat, medium and steep), Substrate of seafloor (coral, algae and sediment). Attention was paid not to include too many criteria and sub-criteria, in order to avoid confusion and reducing lack of focus among interviewees.

Second, we selected survey participants by implementing a non-stratified random sampling from among all households of the island, in the aim of gathering the overall fishers’ spatial preferences and avoiding social-, cultural- and gear-related bias. Overall, 51 coral reef fishers (i.e., household members present at the time of our visit that declared to have fished over the last two weeks) who accepted to participate to the survey (acceptance rate = 96.2%) had a mean age of 32.7 years (min = 12, max = 60, SD = 9.76), were native of French Polynesia (98%) and mostly male (94.6%).

Third, we conducted a survey that included a ranking exercise based on the Analytic Hierarchy Process (AHP) decision-making methodology [55] to measure sub-criteria weights identified in step 1 (S1 File). AHP uses a multi-level hierarchical structure of criteria and sub-criteria that are pairwise compared by participants (here fishers) to derive criteria weights and estimate the consistency of the judgments (called consistency ratio). The difference in importance between each pair of criteria and sub-criteria was indicated by fishers on a 4-point scale (1 = same, 2 = slightly higher preference, 3 = higher preference, 4 = major preference) for which we assigned scores with intervals of three to fit with the 10-points scale used in the AHP methodology [55].

Fourth, we calculated an aggregated weight for each sub-criterion as its average weight among fishers, weighted by the judgment consistency ratio of their response.

Finally, we represented aggregated sub-criteria weights spatially using high resolution maps of each criterion and sub-criterion. Space borne imagery was used along with geolocated acoustic depth measurements and seafloor data, to predict and map Depth, Slope of seafloor and Substrate of seafloor criteria and sub-criteria [69]. Areas where the models did not perform satisfactorily (i.e., where depth exceeded 12m or where the water was turbid) were discarded from the analysis. Using spatial processing, we derived the Distance to pass and Distance to shore criteria and sub-criteria based on coastline and reef crest maps extracted from the same satellite imagery. For each criteria map, every 5 x 5m cell was assigned its corresponding aggregated sub-criteria weight. We then summed all criteria maps to obtain the fishing suitability map (FS), whose cells’ value potentially ranged from a low of 0 to a high of 1. Additional information on the creation of the spatial data can be found in S2 File.

#### Mapping fishing capacity

In Moorea, most coral reef fishers start their fishing trip from the closer access point from their home, but there is no reliable estimate of their number, the intensity of their fishing activity and their location around the island. The number of households and their dependence on marine resources (see below) were thus used as a proxy of the fishing capacity. A dependence on marine resources index, *D*, was calculated for each district (n = 69, Fig 2) from population census data [70] and based on established protocols [71,72] (Eq 1):

$$D=\frac{F}{F+NF}\times \frac{N}{F+NF}\times \frac{U}{N}$$ (1)

where *F* is the number of households having at least one member who declared fishing as its primary or secondary livelihood activity; *NF* is the number of households having at least one member who declared non-fishery-related occupation as its primary or secondary livelihood activity; *U* is the number of households having at least one member having no activity, whether primary or secondary; and *N* is the total number of households. The first term in Eq 1 captures the ratio of fishery-related activities to the overall livelihood activities within the district. The second term captures the extent to which households engaged in fisheries also engage in non-fishery livelihood activities. The third term captures the degree to which livelihood activities determine the subsistence of the other–inactive–members. The second and third terms thus decrease the level of dependence when many households are engaged in both occupational categories, and when inactive people represent a small portion of the district population, respectively. Although it could be argued that the absence of inactive household members in a district (U = 0) may lead to null dependence on marine resources (D = 0)–even in households only engaged in fishing–such configuration does not exist in our data set.

We then mapped the households’ dependence on marine resources by locating each household and assigning them their corresponding district-level level of dependence on marine resources (D). Finally, household density, weighted by the level of dependence on marine resources, was extrapolated onto the lagoon using linear decay to map fishing capacity (FC). The underlying assumptions are that (i) fishing capacity is high in areas with high household density and dependence on marine resources, and (ii) coral reef fishers in Moorea all travel at the same maximum distance from their home. This assumption is reasonable given that fishing trips never exceeded a couple of hours including travel time from home to sea access point. Based on responses given during preliminary interviews with six key informants, we fixed the distance at which fishers could fish at 2 km around individual households.

#### Mapping relative fishing effort

The predicted fishing effort (FE) was finally calculated in each cell according to the following formula:

$$FE_{c}=FC_{c}*FS_{c}$$ (2)

where *FE*_*c*_, *FC*_*c*_ and *FS*_*c*_ are respectively the predicted fishing effort, fishing capacity and fishing suitability at the 5 x 5m cell c.

All statistical and spatial analyses were implemented in the R statistical software version 3.2.2 [73] using the {rgdal} package [74].

### Ethics statement

We followed the Code of Ethics adopted by CRIOBE and validated by the Ethics Committee of the CNRS. Accordingly, fishers involved in the study were informed about the purpose of the questionnaire as well as data use and diffusion. We obtained verbal consent from participants prior to conducting surveys. If provided, we also recorded personal contact information to facilitate restitution of results to participants. Population census data were provided through a memorandum of understanding that CRIOBE has with the *Institut des Statistiques de la Polynésie française* (ISPF) and adhered to the CRIOBE Code of Ethics for research involving people."

## Results

**Fig 3:**
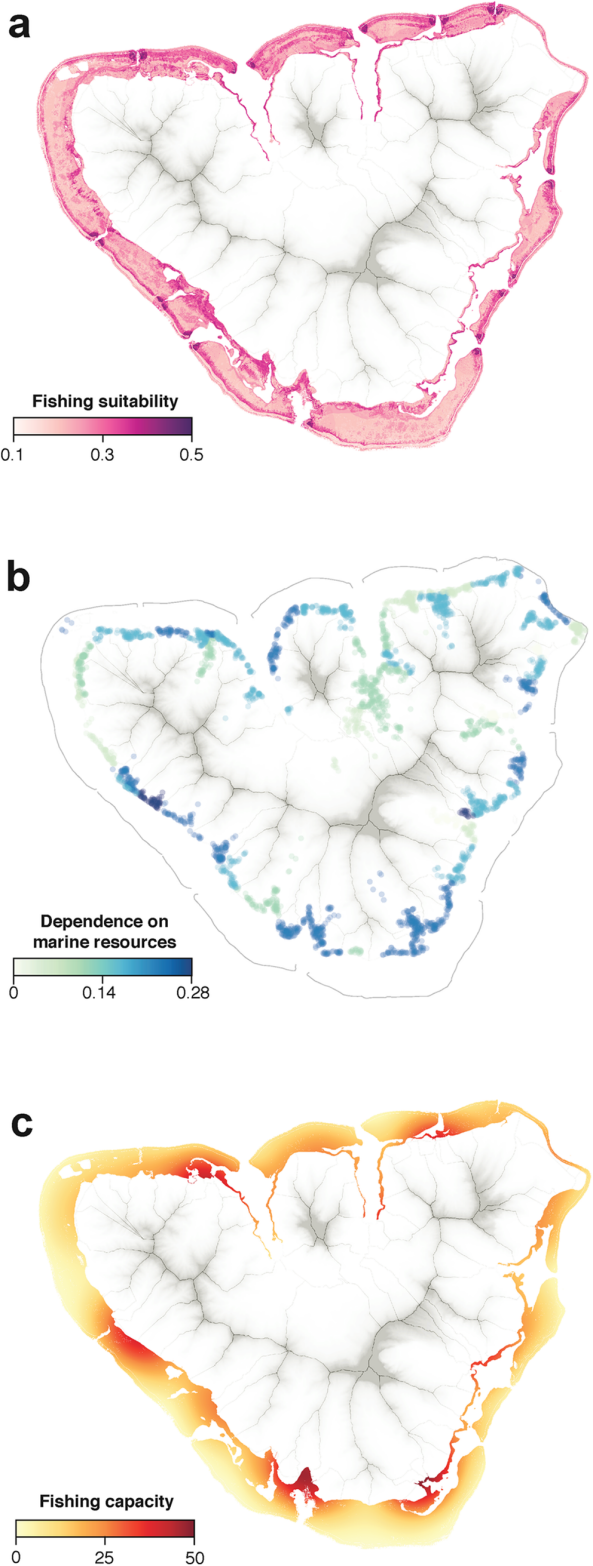
Information used to calculate the two components of the predicted fishing effort: fishing capacity and fishing suitability. (a) Spatial representation of sub-criteria weights measured using the AHP methodology. See S2 File for additional information on the approach used to map sub-criteria weights. (b) Households’ dependence on marine resources. Dots represent households and colors indicate their district-level level of dependence. (c) Fishing capacity, calculated using the cumulated distance to households within a 2-km radius, weighted by their level of dependence on marine resources.

General patterns regarding fishing ground selection were successfully described despite the great diversity of fishing practices and fishers’ profiles. The three sub-criteria preferred by fishers (i.e., greater weights) are coral substrate (0.17 ± 0.03 95% CI), short distance to reef passages (0.13 ± 0.04 95% CI) and steep bottoms (0.09 ± 0.03 95% CI) (Table 1). Because they often combine the highest ranked sub-criteria, reef passages’ edges appear as one of the most suitable fishing ground, despite a general exposure to strong current and high exposure to waves (Fig 3A). Fishers ranked higher sub-criteria generally associated with high fish abundance (e.g., coral substrate, steep rocks) and low travel cost (short distance to the shore significantly ranked higher than large distance). Another important type of fishing area for fishers includes fringing reef covered by hard corals (Fig 3A). Households highly dependent on marine resources for food and/or livelihoods are spread around the island (Fig 3B). The dependency on marine resources island-wide is variable among the 69 districts (Fig 3B) with district-level levels of dependency varying from 0 (Temae, where no household had members engaged in fishing) up to 0.23 (Maatea), 0.25 (Taotaha) and 0.28 (Putoa). Lagoon areas located in front of most dependent populated areas have higher levels of fishing capacity, while remote areas display low levels of fishing capacity (Fig 3C).

**Table 1:** Averaged sub-criteria weights (+/- 95%CI) obtained from AHP exercises performed with local fishers to estimate their preference for fishing grounds. The three sub-criteria ranked higher are indicated in bold.

Criteria	Sub-criteria	Weight +/- 95%CI
**Substrate of seafloor**	**Coral**	**0.166 +/- 0.028**
	Sediment	0.074 +/- 0.023
	Algae	0.029 +/- 0.004
**Distance to reef passage**	**0-250m**	**0.126 +/- 0.038**
	250-1000m	0.041 +/- 0.01
	>1000m	0.026 +/- 0.003
**Slope of seafloor**	**High**	**0.087 +/- 0.026**
	Low	0.057 +/- 0.014
	Medium	0.039 +/- 0.01
**Distance to shore**	0-400m	0.067 +/- 0.029
	400-1000m	0.036 +/- 0.01
	>1000m	0.027 +/- 0.009
**Depth**	3-8m	0.066 +/- 0.014
	>8m	0.038 +/- 0.014
	0-3m	0.038 +/- 0.012

**Fig 4:**
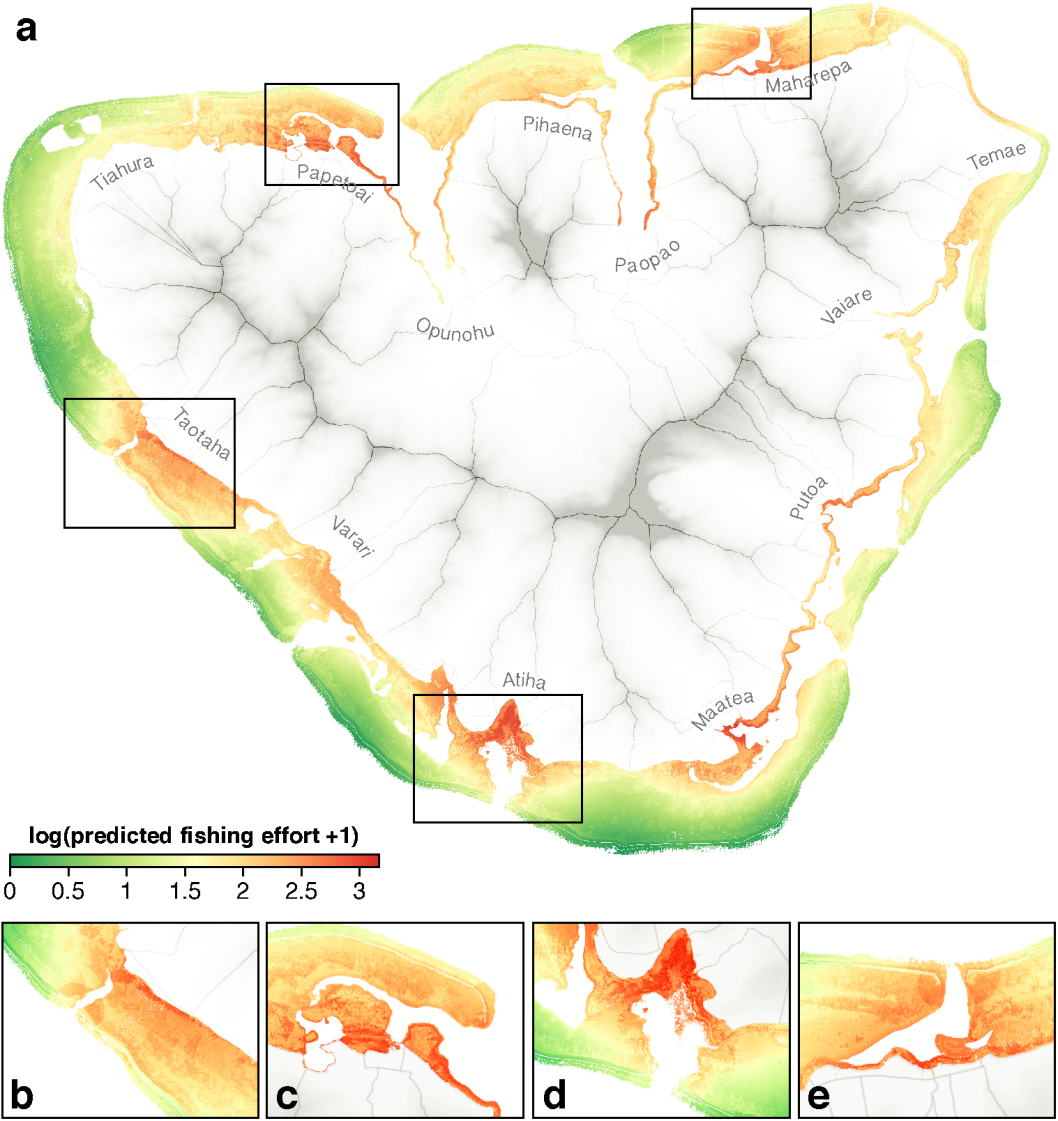
Spatial variation in predicted fishing effort. (a) Island-wide analysis highlights a high level of fine-scale spatial variation in predicted fishing effort. Highly exposed areas include (b) Taotaha, (c) Papetoai, (d) Atiha and (e) Maharepa villages.

On average, predicted fishing effort decreased with distance to coast (mostly due to fine-scale, within-reef habitat heterogeneity) and was variable along the coast (mostly because of varying household density and dependency on marine resources) (Fig 4A). Near-shore areas and reef passages generally displayed relatively higher levels of predicted fishing effort, whilst lower relative levels remained far from the shore (outer reef and remote lagoon areas) and in shallow, flat and less complex areas. Highest levels of predicted fishing effort were found in Taotaha, Papetoai, Maharepa, Atiha (Fig 4B–4E) and on the fringing reef of Maatea. Varari also displayed high levels of predicted fishing effort, while the South point and the North-West side of the lagoon (Tiahura) were among the least exposed to fishing pressure according to our model.

## Discussion

Failure of resource management strategies to achieve the triple bottom line of social, environmental and economic sustainability is often related to the lack of reliable information on spatial patterns of fishing effort [25] and the weak ability (or the lack of opportunity) of key stakeholders (e.g., resource users) to affect the outcome of the decision-making process [75]. The approach proposed here tackle these issues by integrating participatory approaches with socioeconomic approaches, thereby scaling in coverage the spatial distribution of fishing effort in a cost-effective manner.

Associating various stakeholders (i.e., policymakers, managers, scientists and fishers) in the decision making requires a reflexive analysis of the context of production and use of data on fishers’ spatial preference. Direct participatory approaches to map fishing suitability (the first component of the fishing effort) are only well suited when trust among stakeholders is high and power relations balanced, because of three main challenges. The first is ethical, since the consequences of transferring very specific information on fishing areas beyond traditional boundaries (i.e., to science and management) are difficult to control and can be detrimental to resource users [76]. The second refers to the modalities of participation: which people have spoken and what knowledge and techniques are then revealed while others may remain hidden? The third is linked to the formatting of knowledge into databases that can lead local populations to be dispossessed of their knowledge in the profit of development or management ideologies [76,77].

With this is mind, and given the complex context in Moorea, we adopted an indirect participatory approach to quantify fishers’ preference for spatially-explicit criteria. The set of pairwise qualitative comparisons developed in the AHP [55] enabled us to overcome trade-offs made by fishers when making decisions about where to go fishing. Importantly, this process is advantageous as it does not require participants to directly identify specific places of interest, as is the case with direct participatory mapping methods. Rather, respondents are only asked to broadly compare preferred criteria and sub-criteria in relation to each other. This difference in approaches is fundamental because it enables respondents to feel more secure, protecting them from sharing highly sensitive information with strangers (interviewers) and keeping specific fishing grounds a secret. It also avoids biased information related to illegal behavior like poaching. To our knowledge, an AHP analysis has not yet been used directly with fishers to identify key factors driving the spatial preference of fishers, although a related approach was recently applied by Kavadas *et al*. [52] in their study of the Greek artisanal fishing fleet where expert judgment was used.

Based on our experience we suggest that AHP is likely to be a good methodology to investigate spatial preference with fishers, particularly when the initial level of mutual trust is low, when only coarse weight estimation of the criteria that characterize fishing areas are needed and when the required sample would be large (e.g., when fishing practices are highly diverse). In our case, performing such a simple and non-intrusive exercise helped us to establish dialogue, trust and cooperation with interviewed fishers. We therefore believe that this method can contribute to complementing and facilitating the other–more sensitive and difficult to implement, but also more accurate–direct participatory mapping approaches. Besides, contrary to more demanding participatory approaches [78], the AHP approach is simple and standardized enough to be taken up entirely by the local stakeholders (fishers, policymakers and/or managers) and enable continuous, regular and long-term monitoring of the predicted fishing effort. This may in turn enhance participation and increase sample size, ultimately improving spatial preference estimates and allowing the investigation of within-community differences in fishing ground features (e.g., spatial segregation per gender, gear or other factors) and temporal assessments (i.e., seasonal variations) in highly complex fisheries settings.

General patterns regarding fishing ground selection were successfully described despite the great diversity of fishing practices and fishers’ profiles. Exposure to less than favorable conditions (short distance to pass) was ranked second in the overall sub-criteria. Indeed, local fishers have long been aware of the ecological importance of reef passages, which bridge the lagoon and the ocean, are often times important spawning aggregation sites, and may serve an important passage for many fishes that enter the lagoon at dawn to feed during the day and exit at sunset [79,80]. Such features make reef passages of particular interest for experienced fishers (i.e., skilled sailors with good knowledge about currents and lunar cycles).

We obtained fishing capacity (the second component of the fishing effort) through a combination of household density and level of dependence on marine resources. Although aggregated at the district level, areas displaying the highest levels of dependence on marine resources highlighted in this study (Atiha, Maatea, and Taotaha) correspond to the main fishing villages described in the literature [64]. Areas displaying the lowest level of fishing capacity were found in Temae, which is known for being highly embedded into salaried employment and tourism activities [70]. Some intermediate configurations were also identified (e.g., Pihaena and Maharepa). A benefit of considering both household density and dependence on marine resources resides in overcoming conventional approaches that constrained fishing capacity estimation to some particular villages, which limits the generalization of results to surrounding areas. Practitioners conducting fishing capacity assessments at the system-scale (e.g., including several villages or a continuous urban area) are often confronted with a lack of fisheries data and may turn to coarse proxies such as population density to estimate the fishing capacity [42]. The method described here offers a more nuanced view of the fishing capacity, releasing the investigator from major assumptions such as the spatially-homogeneous distribution of fishing households along the coast. Integrating our novel index of dependence on marine resources into household density extrapolation on lagoon waters added a new level of fishing capacity resolution that bridged “coastal population density” and “number of fishers”.

In the context of fisheries management, the lack of information on spatial patterns of resource use makes decisions for marine spatial planning more difficult and potentially at odds with the underlying social-ecological configuration of the system. Fishing capacity roughly indicates where resource extraction is likely to be high (or low), and can therefore fail to provide information fine enough to differentiate areas in patchy and heterogeneous configurations. Combined with spatial preferences (i.e., fishing suitability), it provides insights on the extent to which fishers interact with the marine environment. In our case study, for instance, one might expect the South point of the island to be highly exposed to fishing because it is located close to the fishing villages of Maatea and Atiha. However, with the exception of the fringing reef, this part of the lagoon is mainly composed of shallow sandy bottoms, which are generally of low interest for local fishers and thus shows a remarkably low level of predicted fishing effort. Hence, integrating spatially explicit data on fishers’ preference for fishing grounds (i.e., fishing suitability) adds a crucial level of accuracy in the appreciation of the fishing effort over space, aligning fishing effort mapping within the local context.

Our approach provides relative fishing effort; it does not provide absolute number of boats or biomass caught. While such absolute estimates are more desirable, in their absence, relative fishing effort maps can be used as a systematic decision support tools to represent resource use or opportunity cost (i.e., the cost of management to fishers) [81,82].

Co-construction of fishing effort maps not only inform marine spatial planning, but can also provide a valuable way to engage stakeholders early and continually in the planning process. Individual or collective restitution of research outputs (maps) to interviewees indeed provides a simple and powerful opportunity to develop proximity between fishers and scientists, to strengthen relationship building, and to foster cooperation for future projects, hence contributing to both stakeholder participation and empowerment [83]. Further, an effective stakeholder engagement process will enable a proactive fine tuning of the marine spatial planning process; it has the potential to enhance the reliability of the maps and to promote their acceptance amongst the population. Here we presented the first attempt at quantifying and mapping fishing effort around Moorea. The similar distribution of fishing effort inside and outside marine protected areas (S3 File) indicates that opportunity costs have not been considered when they were designed, which may explain low support among the population (although they therefore represent a random subset of Moorea’s fishing grounds).

## Conclusion

The standardized and flexible approach we developed here can produce baseline spatial patterns of resource use. This baseline information can then be used to establish a dialogue among stakeholders to provide guidance for fisheries management and environmental conservation policies and to initiate standardized temporal assessments of fishing activity when conventional fisheries methods are not suitable. Although we focused on a small-scale fishery operating in a coral reef ecosystem, the portfolio of direct and indirect participatory and socioeconomic approaches we presented makes our fishing suitability capacity mapping framework suitable for other types of fisheries.

## Supporting information

S1 Table List of Moorea’s districts and corresponding dependency on marine resource. High value: high dependency; low value: low dependency. District IDs can be found at http://ispf.pf.
(XLSX)

S2 Table Outputs of the AHP ranking exercise. Code for columns A-O is criteria_subcriteria. Inconsistency score: 0-high inconsistency; 1-high consistency.
(XLSX)

S1 File Survey questionnaire used to quantify fishers’ preference for fishing grounds. The ranking exercise is based on the Analytic Hierarchy Process (AHP) decision-making methodology and enables to measure the importance of sub-criteria weights in fishers’ fishing ground selection.
(DOCX)

S2 File Extended methods. Creation of the spatial information for mapping criteria and sub-criteria.
(DOCX)

S3 File Additional analyses. Predicted fishing effort inside and outside Moorea’s current marine protected areas.
(DOCX)
