# Supplementary material for: Combining participatory and socioeconomic approaches to map fishing effort in small-scale fisheries
Source: PLoS One. 2017 May 9;12(5):e0176862. doi: 10.1371/journal.pone.0176862 (PMC5423602; doi:10.1371/journal.pone.0176862)
Supplement: S1 File — The ranking exercise is based on the Analytic Hierarchy Process (AHP) decision-making methodology and enables to measure the importance of sub-criteria weights in fishers’ fishing ground selection. (DOCX) [file pone.0176862.s003.docx]

**Ranking exercise**

**2.1 Substrate**

*If we only consider seafloor bottom, which criteria is the most important when going fishing in general? 1 - Same importance, 3 - Moderate importance, 5 - Strong importance, 7 - Very strong importance, 9 - Extreme importance*

| Coral | | | | 1 | Algae | | | |
| --- | --- | --- | --- | --- | --- | --- | --- | --- |
| 9 | 7 | 5 | 3 |  | 3 | 5 | 7 | 9 |

| Sediment | | | | 1 | Algae | | | |
| --- | --- | --- | --- | --- | --- | --- | --- | --- |
| 9 | 7 | 5 | 3 |  | 3 | 5 | 7 | 9 |

| Coral | | | | 1 | Sediment | | | |
| --- | --- | --- | --- | --- | --- | --- | --- | --- |
| 9 | 7 | 5 | 3 |  | 3 | 5 | 7 | 9 |

**2.2 Depth**

*If we only consider depth, which criteria is the most important when going fishing in general?*

*1 - Same importance, 3 - Moderate importance, 5 - Strong importance, 7 - Very strong importance, 9 - Extreme importance*

| 0-3m | | | | 1 | > 8m | | | |
| --- | --- | --- | --- | --- | --- | --- | --- | --- |
| 9 | 7 | 5 | 3 |  | 3 | 5 | 7 | 9 |

| 3-8m | | | | 1 | > 8m | | | |
| --- | --- | --- | --- | --- | --- | --- | --- | --- |
| 9 | 7 | 5 | 3 |  | 3 | 5 | 7 | 9 |

| 0-3m | | | | 1 | 3-8m | | | |
| --- | --- | --- | --- | --- | --- | --- | --- | --- |
| 9 | 7 | 5 | 3 |  | 3 | 5 | 7 | 9 |

**2.3 Slope**

*If we only consider slope, which criteria is the most important when going fishing in general?*

*1 - Same importance, 3 - Moderate importance, 5 - Strong importance, 7 - Very strong importance, 9 - Extreme importance*

| Low | | | | 1 | High | | | |
| --- | --- | --- | --- | --- | --- | --- | --- | --- |
| 9 | 7 | 5 | 3 |  | 3 | 5 | 7 | 9 |

| Medium | | | | 1 | High | | | |
| --- | --- | --- | --- | --- | --- | --- | --- | --- |
| 9 | 7 | 5 | 3 |  | 3 | 5 | 7 | 9 |

| Low | | | |  | Medium | | | |
| --- | --- | --- | --- | --- | --- | --- | --- | --- |
| 9 | 7 | 5 | 3 |  | 3 | 5 | 7 | 9 |

**2.4 Distance to coast**

*If we only consider distance to coast, what is the most important when going fishing in general?*

*1 - Same importance, 3 - Moderate importance, 5 - Strong importance, 7 - Very strong importance, 9 - Extreme importance*

| 0-400m | | | | 1 | > 1000m | | | |
| --- | --- | --- | --- | --- | --- | --- | --- | --- |
| 9 | 7 | 5 | 3 |  | 3 | 5 | 7 | 9 |

| 400-100m | | | | 1 | > 1000m | | | |
| --- | --- | --- | --- | --- | --- | --- | --- | --- |
| 9 | 7 | 5 | 3 |  | 3 | 5 | 7 | 9 |

| 0-400m | | | | 1 | 400-1000m | | | |
| --- | --- | --- | --- | --- | --- | --- | --- | --- |
| 9 | 7 | 5 | 3 |  | 3 | 5 | 7 | 9 |

**2.4 Distance to reef passages**

*If we only consider distance to reef passages, which criteria is the most important when going fishing in general? 1-Same importance, 3-Moderate importance, 5-Strong importance, 7-Very strong importance, 9-Extreme importance*

| 0-250m | | | | 1 | > 1000m | | | |
| --- | --- | --- | --- | --- | --- | --- | --- | --- |
| 9 | 7 | 5 | 3 |  | 3 | 5 | 7 | 9 |

| > 1000m | | | | 1 | 250-1000m | | | |
| --- | --- | --- | --- | --- | --- | --- | --- | --- |
| 9 | 7 | 5 | 3 |  | 3 | 5 | 7 | 9 |

| 0-250m | | | | 1 | 250-1000m | | | |
| --- | --- | --- | --- | --- | --- | --- | --- | --- |
| 9 | 7 | 5 | 3 |  | 3 | 5 | 7 | 9 |

**2.5 Criteria**

*All criteria considered together, which is the most important when going fishing in general? 1 - Same importance, 3 - Moderate importance, 5 - Strong importance, 7 - Very strong importance, 9 - Extreme importance*

| Depth | | | | 1 | Dist shore | | | |
| --- | --- | --- | --- | --- | --- | --- | --- | --- |
| 9 | 7 | 5 | 3 |  | 3 | 5 | 7 | 9 |
|  |  |  |  |  |  |  |  |  |

| Depth | | | | 1 | Dist pass | | | |
| --- | --- | --- | --- | --- | --- | --- | --- | --- |
| 9 | 7 | 5 | 3 |  | 3 | 5 | 7 | 9 |

| Depth | | | | 1 | Slope | | | |
| --- | --- | --- | --- | --- | --- | --- | --- | --- |
| 9 | 7 | 5 | 3 |  | 3 | 5 | 7 | 9 |

| Depth | | | | 1 | Substrate | | | |
| --- | --- | --- | --- | --- | --- | --- | --- | --- |
| 9 | 7 | 5 | 3 |  | 3 | 5 | 7 | 9 |

| Dist shore | | | | 1 | Dist pass | | | |
| --- | --- | --- | --- | --- | --- | --- | --- | --- |
| 9 | 7 | 5 | 3 |  | 3 | 5 | 7 | 9 |

| Dist shore | | | | 1 | Slope | | | |
| --- | --- | --- | --- | --- | --- | --- | --- | --- |
| 9 | 7 | 5 | 3 |  | 3 | 5 | 7 | 9 |

| Substrate | | | | 1 | Dist shore | | | |
| --- | --- | --- | --- | --- | --- | --- | --- | --- |
| 9 | 7 | 5 | 3 |  | 3 | 5 | 7 | 9 |

| Slope | | | | 1 | Dist pass | | | |
| --- | --- | --- | --- | --- | --- | --- | --- | --- |
| 9 | 7 | 5 | 3 |  | 3 | 5 | 7 | 9 |

| Substrate | | | | 1 | Dist pass | | | |
| --- | --- | --- | --- | --- | --- | --- | --- | --- |
| 9 | 7 | 5 | 3 |  | 3 | 5 | 7 | 9 |

| Substrate | | | | 1 | Slope | | | |
| --- | --- | --- | --- | --- | --- | --- | --- | --- |
| 9 | 7 | 5 | 3 |  | 3 | 5 | 7 | 9 |

1. **Additional remarks**

……………………………………………………………………………………………………………………………………………………………………………………………………………………………………………………………………………………………………………………………………………………………………………………………………………………………………………………………………………………
