# Supplementary material for: Combining participatory and socioeconomic approaches to map fishing effort in small-scale fisheries
Source: PLoS One. 2017 May 9;12(5):e0176862. doi: 10.1371/journal.pone.0176862 (PMC5423602; doi:10.1371/journal.pone.0176862)
Supplement: S2 File — Creation of the spatial information for mapping criteria and sub-criteria. (DOCX) [file pone.0176862.s004.docx]

**Creation of the spatial information for mapping fishing suitability**

Satellite data was used along with acoustic depth measurements and seafloor data, to respectively predict depth and habitat composition at any 5 x 5m pixel of the lagoon (see details bellow). Areas where the models did not perform well (i.e., deep areas and/or turbid areas) were removed from the analysis. Coastline and reef crest were also extracted from the satellite image using GIS procedure. Five criteria, each divided into three sub-criteria, were finally identified following preliminary interviews with fishers and represented spatially based on the digital spatial models described above (Figure 1-6).

**Figure 1: Flow diagram illustrating the process used to create the spatial information used to map fishing suitability.** Details are given bellow for each criteria.

**Depth** was mapped using a combination of accurate sonar soundings and continuous spaceborne imagery. A total of almost 16,000 soundings spanning the first 20 m water depths was collected in January 2011 with a small boat provided with a combo 200-kHz echosounder / 12-channel GPS receiver. In parallel, a very high resolution Pleiades-1 imagery composed of four multispectral bands (blue, green, red and near-infrared with 2-m pixel size) and one panchromatic band (0.5 m pixel size) was acquired on 23 June 2014. Following geometric, radiometric corrections and pansharpening procedure (see Collin and Hench 2015 for further details), the 0.5-m visible multispectral dataset was used to train and validate a neural network model standing for the bathymetric model. Very high agreements (r=0.89, R^2^=0.8 and RMSE=2.44 m) between predicted and actual ground-truth were found out so as to build a digital depth model (DDM) of Moorea bound by 0 and 20 m. The DDM was resampled at 5-m spatial resolution and turbid areas or areas below 12 meters (19.5 % of the total reef area), were removed from the analysis because of the growing uncertainty with depth regarding the substrate (Figure 2).

**Figure 2: Maps of “depth” criteria.** (a) Continuous scale. (b) Categorized according to the sub-criteria boundaries.

**Distance to the shore** was calculated in meters and expressed as the minimum distance of each 5 x 5 m pixel to the nearest coastline (Figure 3). This criterion expresses the minimum distance fishers must travel to fish in this pixel. Since the fore reef in only accessible by the passes, we used the r.cost.full function in GRASS to force the distance calculation to account for the reef crest.

**Figure 3: Maps of the “distance to shore” criteria.** (a) Continuous scale. (b) Categorized according to the sub-criteria boundaries.

**Distance to the pass** was calculated in meters and expressed as the minimum distance of each 5 x 5 m pixel to the nearest pass (Figure 4).

**Figure 4: Maps of the “distance to pass criteria”.** (a) Continuous scale. (b) Categorized according to the sub-criteria boundaries.

**Substrate** were mapped using a synergy of punctual photoquadrats and seamless spaceborne imagery. An array of 897 geolocated ground-truth pictures were gleaned from June 2010 to November 2011 using a small boat adapted to navigate in very shallow waters. Following a thorough examination of the pictures, a gamut of 22 benthic classes were discriminated so as to categorize the pictures. The spectral signature of each of the 22 classes was computed using the associated pixels contained into the corrected 2-m multispectral Pleiades-1 dataset. A very high coefficient of agreement (kappa=0.91) was derived from the confusion matrix. Because of the straightforwardness of this paper objective, we merged the 22 classes into 4 super-classes: deep water, sediment, algae, hard corals (Figure 5). Deep water pixels were removed (see Depth). Due to the uncertainty regarding substrate sub-criteria, we calculated each pixel’s score using the following formula:

$S= \frac{S_{C} \times P_{C}}{100}+ \frac{S_{A} \times P_{A}}{100}+ \frac{S_{S} \times P_{S}}{100}$ (Eq. 1)

where S_C_, S_A_ and S_S_ are sub-criteria scores for coral, algae and sediment, respectively; PC, PA and SS are the probability of the substrate to be to encounter coral, algae and sediment in %.

**Figure 5: Maps of the “substrate” criteria.** Probability of the substrate to be (a) coral, (b) algae and (c) sediment.

**Slope** was calculated in degree and expressed as the greatest drop in bathymetry among the 8 neighboring pixels. We computed the slope based on the bathymetry resampled at 20-m resolution is order to account for slope variation rather than undesirable fine scale variation in slope that could have arisen from the presence of numerous bommies or error in the bathymetry model (see Depth section).

**Figure 6: Maps of the “slope” criteria.** (a) Continuous scale. (b) Categorized according to the sub-criteria boundaries.

**Reference:**

Collin A. and Hench J. 2015. Extracting shallow bathymetry from very high resolution satellite spectral bands and a machine learning algorithm. *International Council of the Exploration of the Sea (ICES)* CM/N:24.
