# Supplementary material for: Combining participatory and socioeconomic approaches to map fishing effort in small-scale fisheries
Source: PLoS One. 2017 May 9;12(5):e0176862. doi: 10.1371/journal.pone.0176862 (PMC5423602; doi:10.1371/journal.pone.0176862)
Supplement: S3 File — Predicted fishing effort inside and outside Moorea’s current marine protected areas. (DOCX) [file pone.0176862.s005.docx]

**Predicted fishing effort inside and outside Moorea’s marine protected areas**

**
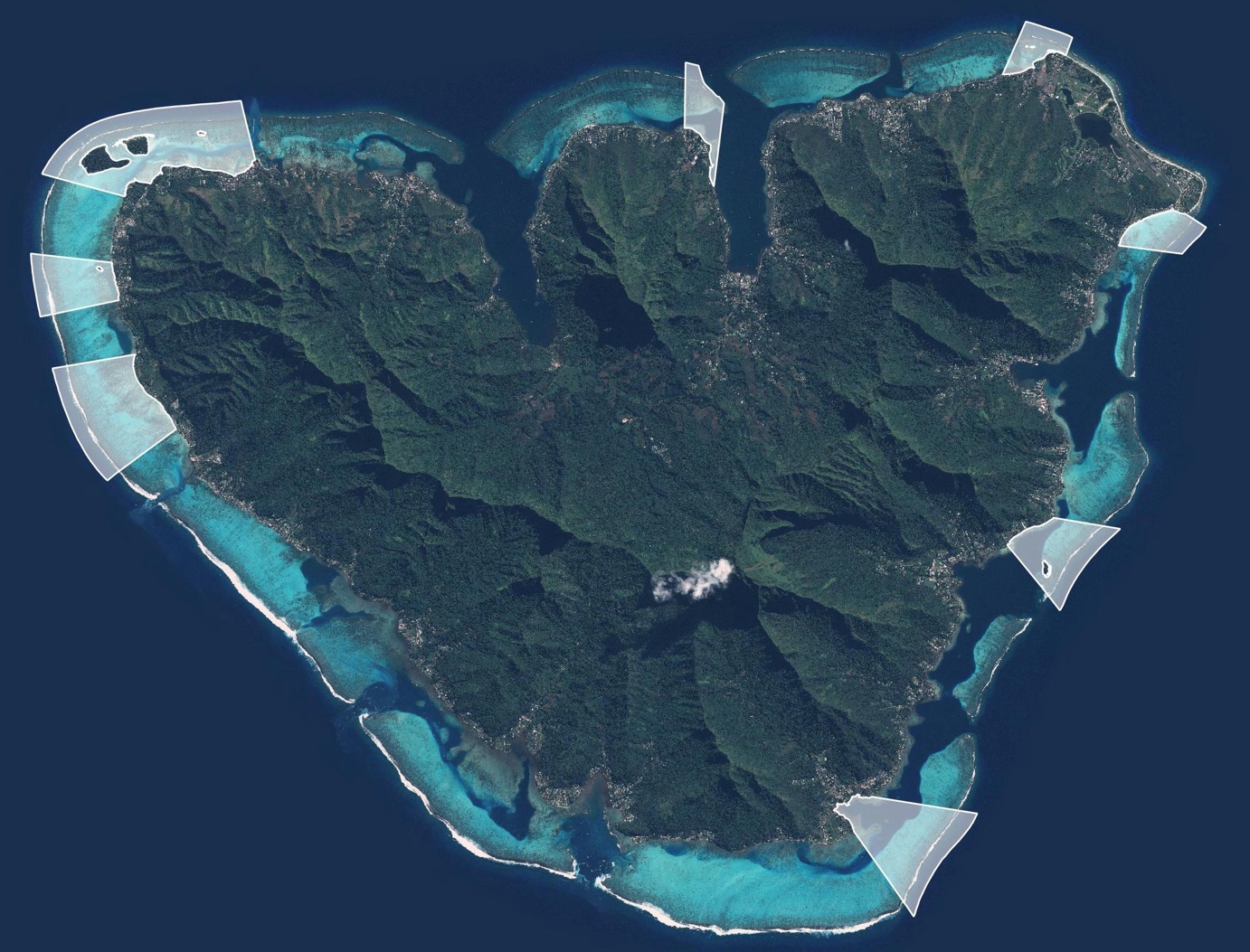
**

**Figure 1: Map of Moorea showing the location of the eight marine protected areas.**

**
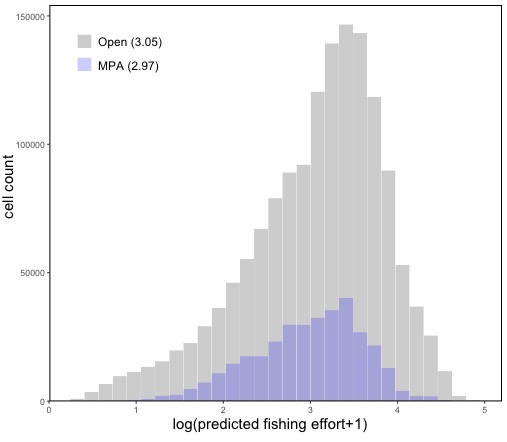
**

**Figure 2: Distribution of predicted fishing effort inside (MPA) and outside (open) marine protected areas (means in parentheses).**
